# Ohio State Dental Society

**Published:** 1898-03

**Authors:** 


### THE DENTAL REGISTER.

Vol. LII.] MARCH, 1898. [No. 3.

### PROCEEDINGS.

### Ohio State Dental Society.

(Continued from Feb. No. page 69.)

SECOND DAY—MORNING SESSION (*Continued.*)

Dr. H. T. Smith, of Cincinnati, read the following paper:

THE COAGULATION THEORY.

Not long since I came across the following statement in the editorial pages of the *International Dental Journal.* It is headed, "The Coagulation Theory," and reads, "The discussion on this subject, which has taken a wide range and claimed the interest of many minds for years, seems to be drawing to a conclusion, if the very thorough experiments of Dr. E. L. York are to be accepted. These investigations clearly prove that a coagulant such as carbolic acid does diffuse through dentin, notwithstanding assertions made to the contrary, and does not form an impenetrable coagulum at the orificial ends of the dentinal tubuli. These investigations substantiate those previously made, and, conjoined by those made by Dr. Bethel with silver nitrate, form a series of facts that clearly settle the question." I thought it might be interesting to look up the points at issue and to forecast if possible the conclusion spoken of, and at the same time call attention to some points in the therapeutics of root-canal treatment as they occur.

The question originally at issue in the coagulation theory was whether or not a medicament which has coagulating properties, if used in the treatment of root-canals, so seals the mouths of the dentinal tubes as to prevent the further ingress of itself or of another medicament afterward used for antiseptic treatment? In other words, are carbolic acid and zinc chlorid self-limiting in their action, and, if so, are they contra-indicated in the treatment of root-canals? It involves also this question, which is quite as important: Is the product matter from a putrefactive pulp capable of entering the body of the dentin, and, if so, is it also capable of infecting the pericemental tissues, causing what we recognize as lame teeth?

Looking into the first question, the diffusibility of coagulants, Dr. Harlan's experiments were probably the first made, and were about as follows: The pulps of freshly-extracted teeth were removed, the ends of the roots sealed, and the teeth planted in plaster of Paris. Cotton moistened with carbolic acid in the one case, and with wood creosote and zinc clorid in the others, was carefully packed in the root-canals. At the end of forty days tests were made in the plaster of Paris for the presence of the coagulant, and none was found. When the essential oils, cloves, cassia, and eucalyptus were used, it was found after four hours that the oils had plainly penetrated the dentin. By adding carmin to the oils the degree of penetration was distinctly followed. When solutions of silver nitrate were used it was found not to penetrate the tubules to any extent, and it was classed as self-limiting with the other coagulants. The silver nitrate furnishes its own stain, and the degree of penetration was easily defined in sections of the roots. In another set of experiments Dr. Harlan immersed the teeth in starch solution up to their anatomical necks, after treating the canals with coagulating disinfectants and iodin. The characteristic blue color did not appear in the starch solution, hence he concluded that the coagulants were a bar to their own diffusibility. We cannot but believe that these experiments were carefully made and the results accurately reported.

The other side of the question is best represented by the recent experiments of Dr. E. L. York upon the diffusibility of coagulants in dentin, to which the editorial quoted refers as those that settle the question.

Using a number of teeth with both normal and putrescent pulps, Dr. York prepared them about as Dr. Harlan did. He injected into the canal of one a ninety-five percent carbolic acid, which had previously been colored with a small quantity of fuchsin. The tooth was sealed and wrapped in moist gauze, and was kept at a temperature of 98° F. to simulate the conditions in the mouth. In about eighteen hours the carbolic acid had passed through the dentin, as shown in sections of the root. This experiment was repeated a number of times, so that no doubt as to the result was left in the experimenter's mind. In other experiments the teeth were suspended in water and the test for carbolic acid made with bromin water, when it was found that the carbolic acid had penetrated the roots in about eighteen hours. Next, in order to demonstrate that carbolic acid does not form an impenetrable coagulum at the orificial end of the dentinal tubuli with their albuminous contents, one of the teeth that had contained carbolic acid in its root-canal for seventy hours was used. The canal was dried and the saturated solution of sodium chlorid was sealed in it. The tooth was suspended in water, and after three hours the water was tested for the presence of the sodium chlorid, using a drop of silver nitrate. The result showed a quantity of silver chlorid thrown down, proving that the coagulum formed was not a barrier to the passage through the dentin of other medicaments used in root-canal treatment. He found also that carbolic acid placed on the white of a boiled egg shows marked diffusibility, from which he infers that carbolic acid is not self-limiting in its action, but penetrates its own coagulum.

Now, here are two sets of similar experiments with exactly opposite results. Which are we to believe? Some one says that it is a question of veracity, but it seems to me that the always possible differences in the dryness and age of the teeth used, and the variations in the quantities and percentages of the solutions used, should be given full consideration in the results. Dr. Truman says the question is settled in favor of the diffusibility of coagulants Hence it is still good practice to use carbolic acid in root-canals. There is, however, one point that the experiments do clearly show, and in this they have perhaps chief value. It is said that the essential oils are very much more diffusible than the coagulants, and for this reason they have a distinct advantage over the latter in certain instances of root-canal treatment; and perhaps the mistake is made when the question is not allowed to rest with this comparison, but is pushed to the ostracism of one or the other class of medicaments.

A few observations I have put down as they occurred in looking up this question. The first is the statement of Dr. Bethel about the use of silver nitrate. If applied without the use of cataphoresis it is practically self-limiting, and even with the aid of cataphoresis there is little or no danger of forcing the silver nitrate through the whole extent of the dentinal tubules and causing injury to the peridental membrane.

Another observation is the statement that capillary attraction does not come to our aid, as had been supposed, in the rather difficult operation of forcing chloro-percha and medicaments into closed tubes such as are most root-canals. Experiments show that capillary attraction only takes place in minute tubes that are open at both ends; therefore, the solubility of chlora-percha in oil of cassia and other of the essential oils is certainly to be taken advantage of by treating the canal, just previous to filling with one of these oils.

Another observation is the radical statement I found, that the successful treatment of pulpless teeth depends first upon the exclusive use of diffusible disinfectants, and secondly upon the repeated and continued application of the disinfectant dressing for a considerable length of time. This statement excludes the use of carbolic acid, mercuric chlorid and other antiseptics, and opposes the accepted custom of immediate root-filling, which is certainly good practice in many cases. And, finally, in regard to the future of root-canal treatment, I have only to say that whether or not the advances will be in the line of mummifying pastes or chemico-metallic methods, or will remain a process improved along the present lines of practice—that of dragging out each particle of nerve-tissue to the end of the root and the end of the hour—it is difficult to say, but it is sincerely to be hoped that the improvements will be effected in the methods as well as in the medicaments.

DISCUSSION.

Dr. L. P. Bethel, Kent, said the subject of the paper should receive more attention than it has done, on account of the importance of exact knowledge to the profession. Those who have given it thought should receive credit for their work. What Dr. Truman had said about his (Bethel's) work was not correct. His experiments had been carried on for the purpose of determining the effect of nitrate of silver on the tissue, and not to find the effect of coagulation; he had made no experiments along that line, but thinks there is a good field for work in that direction. The experiments which have been made have been made honestly and carefully, but different experimenters have worked under different conditions, and so the results have varied greatly. Had the work been done under similar conditions, and the same solutions been used, the results would probably have been identical. The conditions are not the same when the tooth is extracted as when it is in the mouth. The experiments should be made on the tooth in the mouth, and then the tooth should be extracted and tested with various color tests to prove the extent of the coagulation.

As to the statement of the paper in regard to the use of nitrate of silver, it is true that a simple application will not penetrate to any great depth; but with cataphoresis for five minutes you will get a decided penetration, and with greater current and more time much deeper penetration. He believed that there was electrolytic action upon the silver nitrate, and that it was converted into its elements and again reunited when driven into the membranes.

Dr. H. A. Smith, Cincinnati, said that he understood Professor Taft to say that when the pulp was removed from the tooth, the life of the tooth was entirely destroyed.

Dr. Taft. It usually is.

Dr. Smith said that the word "usually" knocked his objection out. According to the teaching of the New York School of Histology, there is a reticulum of living matter in the dentin that preserves the life of the tooth. He believed that the pulp should be preserved when it was possible, but Dr. Ingersoll says that with a matured tooth it does not make much difference. For himself, however, he believes with Heitzmann that the living reticulum is distributed widely through the tooth, and does preserve its life. He did not think the careful use of arsenious acid was dangerous—the danger is from using too much. Dr. Hugenschmidt, of Paris, has mentioned a number of cases of alveolar abscess between the apex of the tooth and the neck. This, if true, proves that there must be connection between the tubuli of the teeth and the dead pulp, and we must recognize the fact that the fibrillæ of the teeth distribute nutriment in the tooth-substance.

He did not see any particular advantage in the use of asbestos as described, and thought cotton or other substances would do as well to carry the medicaments.

As to antiseptics, there are many in use by different dentists, all of which, or most of them, apparently answer their purpose satisfactorily. Some dentists depend on one only, and others have several favorites.

Dr. W. I. Jones, Nelsonville, thought it looked as if the whole system of dentistry was based upon the coagulation theory, but he could not understand, if this was so, how it was that so many different antiseptic preparations would work successfully. He did not believe that tests made outside of the mouth had any bearing on practical results. We know that silver nitrate will will make a coating on the dentin that will protect the tooth; but if this coating is burred off, the tooth will be as sensitive as before. If we are to revise the practice of our profession in accordance with the results of these new investigations, it will be necessary that the investigators should be so thorough that their results will not be open to any doubt or uncertainty.

Dr. F. W. Sage, Cincinnati, said that neither the use of silver nitrate nor of asbestos was original, but the combination of the two was original. The silver nitrate has the objection that it discolors the tooth. He thought that the application of sodium chlorid or common salt would lessen this action, especially if cataphoresis was used to drive it into the tubules. This use of sodium chlorid has not, so far as he knows, been referred to by any one. It would be much better it the profession generally would have a uniform practice in the treatment of root-canals, for then when a case presented for treatment one would know what to expect to find at the apex of the root and what to do with it. We do know, however, that when we drill into a dead root for the purpose of placing a crown that we are liable to stir up a hornet's nest. This is because the spores or other effects of the former decomposition have not been fully removed, and when the drill lets air into these bacteria they are worked up and restored to a state of activity.

In a prize paper recently published it is shown that it is necessary to open up the tooth so thoroughly that every particle of septic matter shall be removed. If we can demonstrate that the use of silver nitrate will enable us to drill up into the root of a tooth without causing trouble, we shall have done a good service to the profession.

Dr. C. R. Butler, Cleveland, said: Suppose Dr. Sage or some one else should prescribe a method that would be accepted by this body; how many would use it in their practice? There are nearly as many modes of procedure as there are persons in practice, and the hope of uniformity is very distant. It would be a very good thing if such a recommendation could be followed, as it would leave less uncertainty about how a root had been treated, but as a rule we are so wedded to our own ways that we would not leave them.

In regard to the paper of Dr. Smith, it is a very concise review, with some little filling in of the views of the men who have been accepted as authorities on the subjects of coagulants and the preparation of cavities and chambers of pulpless teeth. He was glad that so young a man had given us such a concise presentation of the whole subject, and we should accept this paper as a good basis for continued work in these lines of investigation. The results of the experiments made by different workers vary so much that their experiments must have been made under different conditions.

The idea that the clearing out of the pulp will end all trouble is like that of the surgeon who believes that to cut off a damaged limb would be the best way to treat the injury. Such radical surgery is not allowable if the limb can be saved; and so with the pulp of the tooth—it is part of the organization and should be preserved if possible.

Dr. J. Taft, Cincinnati, said that his statement was that if the pulp was destroyed the life of the tooth was usually destroyed sooner or later, but he did not say that the tooth immediately became a dead tooth. Sometimes the life of the dentin continues for years, but often it is lost soon after the extermination of the pulp. Of course, the tooth, though dead, may be, and often is, useful for years.

Dr. Henry Barnes said that Dr. McLean used silver nitrate and the oil of cassia. In such cases the silver nitrate was not necessary; the oil of cassia alone is better. In posterior teeth and those with tortuous canals nothing is better than silver nitrate for rendering the canals of a dead tooth antiseptic, but in the front teeth the discoloration which it causes is a fatal objection.

Dr. L. L. Barber, Toledo, asked Dr. Bethel whether he did not consider the penetration of silver nitrate, used either with or without the electric current, to be due to the formation of new compounds with the contents of the tubules.

Dr. Bethel said he had not satisfied himself on this point, but thought that perhaps it was to a certain extent.

Dr. H. E. Dunn, Warren, thought it very necessary to use dehydrating apparatus to dry the root before other treatment; recommended Evans' "root-drier" for the purpose.

Dr. McLean said he had had but about ten years of practice and was not skillful enough to cap an exposed pulp and preserve it. He usually removed it with the use of ethyl-chlorid spray. He believed that the more dentin is removed from the root-canal the better, as there is less septic material remaining; and when filled there is more probability of preserving the tooth. He did not think pulps could be removed as well by cocain as by ethyl chlorid, as the latter anesthetizes the pulp so thoroughly that there is no pain.

As to the variations in pulps and in the direction and construction of the canals, there are no two teeth alike, and we have to use our intelligence to recognize the actual conditions. He did not believe in the use of acids, as their action can not be limited; the silver nitrate is self-limiting. The reason that asbestos is better for filling root-canals than other material is because the powder is so fine that we can get it to any point where a broach can be made to enter. Some one said that it was not necessary to place the rubber-dam in position when about to remove the pulp. In reply to this it is enough to say that it is impossible to find an aseptic mouth; not to place the rubber-dam will be to invite the entrance of septic matter into the canals, and would be an inexcusable act of carelessness on the part of the operator. As to the various modes of treatment, he did not care for the experience of others; what he wanted to know was what he could best do himself for the benefit of his patients.

Dr. H. T. Smith, in closing the discussion, said that when the subject of treatment of root-canals was up for discussion before a local society in Cincinnati it had been decided that the canals were generally over-treated, and that when there was after-trouble it was generally because of the condition of the tissues beyond the root-canal.

The subject was passed, and Dr. W. D. Snyder, of Sidney, read the following paper:

I wish to say that I was not invited to read a paper before this convention, but was informed by the committee that I was expected to do so, and as a member of this honorable body I felt it my duty to make an effort. This, coupled with the fact that the subject with which I shall try to deal has always been of great interest to me, is my excuse for occupying a few minutes of your valuable time. The committee did me the kindness, however, to allow me to give the title to my paper, which, as you see by the programme is,

"THAT TROUBLESOME NERVE," OR PAINLESS DENTISTRY.

The practice of dentistry has many perplexing questions, and is beset by many annoying criticisms. In the operating department there is no one thing that causes more worry, or tends to make prematurely gray those once raven locks, than that little anatomical mass of odontoblasts, blood-vessels, white and gray matter, commonly known as the "nerve of the tooth." It has been a theme for study during ages past by odontologists, histologists and pathologists, college professor, and student. It seems to me uppermost in the mind of the patient as she sits in the operating chair. It is talked of in the home, at the club, and on the street; in fact, while it can not strictly be said to be a figure in the public eye, it evidently is very much in the public mouth. It has many peculiarities and queer idiosyncrasies. Its lesions are many, and often difficult of diagnosis and uncertain of prognosis. Those lesions may arise from scores of different causes and the symptoms remain about the same. Just when we make up our mind that a pulp is so protected that it will certainly live, it will surprise us by passing suddenly into the great unknown. And after we have spent days and even weeks in trying to destroy its frail existence we find it still very much alive, and we may quote, "How many now are dead to me that live to others yet," etc. Different from most things according to the laws of nature, death does not always end the trouble with this little disturber of the household peace and professional friendship, but goes right on until it is wiped out of existence, literally torn from its habitation, its house thoroughly cleansed and disinfected, and a new tenant moves in that will be more compatible with the surrounding tissue. In dealing with a dead pulp it often requires more skill and better judgment than with one in its normal condition, for very often we find the mere opening of a pulp chamber containing a decomposed nerve results in peridental inflammation and perhaps abscess. Nerve broaches here, either smooth or barbed, should be used with great care, even though the apex be not penetrated by the broach; too much cotton wound upon it might act as a piston, thus forcing enough septic matter or driving enough of the gases beyond the apex to irritate the surrounding parts sufficiently to bring about very bad results. I somewhat question the use of hydrogen peroxid or pyrozone in such cases, especially if left plugged too tightly in the cavity; a more mild dressing of some good antiseptic would be preferable. Again, such cases are not always treated a sufficient length of time, and the roots are filled in a partially septic condition with equally unfavorable results.

There has been a great deal said and much written regarding the filling of root-canals. There are certain rules which must be observed, after which almost any material or method may be employed with a reasonable assurance of success. I have already alluded to the necessity of putting the tooth or root in the very best possible condition. The root-filling should be non-irritating, and one that can be inserted easily and perfectly to the apex.

A very valuable quality for a root-filling to possess is ease of removal, in case of pain or inflammation requiring it. I remember reading an article not long since wherein the writer rather criticised the use of any antiseptic agent in a root-filling, claiming that such properties would sooner or later pass away, thus leaving the root in no better condition than though it had not been inserted. I am of the opinion that the use of an antiseptic in a root is of great value in most cases until after the parts about the apex, where the pulp has been dissected or torn away, have returned to their normal condition. Then it makes no particular difference if these properties do disappear, so the filling of the root remains. My method of filling root-canals is with common lime, mixed with water to a pasty condition with enough carbolic acid and iodoform well mixed with it to give it as much antiseptic and disinfecting qualities as possible. I have used this method for over three years and find it very satisfactory. As root-canal treatment and filling is not the subject of this paper altogether, we will pass to other conditions of "that troublesome nerve" that may have a claim upon some of our time.

There is no operation in which error can more easily be made than in filling over a live pulp. There are always two points to be attained—viz, a good, substantial filling, for that is what is expected of us, and one which will not only guard the pulp against irritation, but will of itself be non-irritating. To be able to excavate and fill a cavity without pain is very desirable, but within a fortnight to have the tooth begin to throb and jump is somewhat embarrassing, and results in a step backward in our very much desired popular dental education regarding the care of the teeth. No cavity, however small, should be filled without first having a coat of some good resinous varnish or non-conducting tooth-lining. Capping an exposed or a wounded nerve is attended with doubtful results, however practiced. We have no evidence that a wounded pulp possesses recuperating or healing power to any available degree, therefore we should be very careful in our operations lest in trying to avoid giving our patient pain we throw open the gates to predisposing causes and let in the very object we would shut out.

It is not the purpose of this paper to dwell at length upon the details of every-day scenes or to enlarge upon relations we, as dentists, bear to the public, nor are we prepared to give to the profession a panacea for all the ills of this troublesome nerve, but we are here to offer what we have gleaned from observation and experience and to open for discussion a subject, though it be old, that should be of great interest to us all. The writer thinks he has good reason to believe that some conditions connected with operations involving this troublesome member might be bettered, that popular dental education could be further advanced, that the dread of the dental chair and unfriendly criticisms largely diminished, if more thought and care were given to such operations.

Gold cap-crowns or large pieces of bridge-work are often placed upon stubs or badly-decayed teeth with portions of pulp-tissue remaining that should be removed; pivot teeth are often set upon roots from which all the pulp has not been removed; or, if so, the root has not been put in the best possible condition. What is the result? Inflammation, pain, swelling, abscess, and a tirade of abuse heaped upon dentists and dentistry in general. There is only one way out of this trouble—cataphoric appliances, arsenious acids, nerve broaches, cotton and medicaments of all kinds, good and bad, are on the market plenty and cheap. Select the instruments and medicine with which you succeed best, and work intelligently.

Is this an age of painless dentistry? I think not. Yet I am glad to note the fact that a great effort is making in that direction. That dentistry is progressing there can be no doubt. Methods, means and medicaments of no small value are each year brought out that redound to the benefit of both the public and the profession. The teachings of our colleges seem to be keeping pace with the times. The terms have in most cases been lengthened, the number of terms increased, and the faculty groomed and coached until they seem to be almost bubbling over with knowledge. Our dental literature, of which there is a fair supply, is teeming with good things. The signs then certainly point to the time when our fair patients will enter our sanctum, arrayed in smiles instead of frowns, and depart with nods and bows instead of groans and tears.

Unfortunately this dreamed of time has not yet arrived, but we may approximate as nearly as possible that golden age by using to the best advantage methods and means within our grasp.

AFTERNOON SESSION.

The convention was called to order at 2:00 P. M.; Dr. Custer in the chair. After transacting some routine business, the discussion of Dr. Snyder's paper was taken up.

DR. HUNTER said he understood that the general trend of the papers this morning was upon the same subject. The paper dealt with facts which confront us, and it is the dealing in these facts which make us a profession. He read a letter from Dr. Wright in which he said, if it were not for "that troublesome nerve" the profession would not have entered the domain of pathology, anatomy or histology. It is the index that points us to the living organisms, and makes it possible and desirable for us to study it closely. I am sure your paper will contain facts of importance and theories for correct practice, and regret that I can not be with you to open the discussion.

DR. W. T. BORN, Kenton, had concluded that there were many things he did not know, and how to cap a pulp successfully was one of them. He prefers to devitalize; it was more safe.

DR. H. L. AMBLER, Cleveland, said that the question, "Was it alive or dead?" put him in mind of a joke the Irishman got off. He said, "If he could breathe without his breath he would not be dead," and if the tooth could live after the pulp was gone it was not dead. The idea that seemed to be expressed this morning was that the aim of the silver nitrate application was to stop off the canal at the apex of the root, but the truth is it coats the whole canal. One gentleman spoke of using one to two grains of arsenious acid for devitalizing the pulp. The truth is one-fiftieth of a grain is as much as should have been used, and it should never be used in deciduous teeth. The acme of methods of treating pulps and canal is the nitrate of silver treatment.

Dr. H. E. Dunn, Warren, said there seemed to be uncertainty as to the action of arsenious acid on the teeth. How does it act; by limited escharotic effect? He had heard it said it produces a strangulation at the apex; the fact that a portion of the pulp remaining alive proves that it is by escharotic action; it does not penetrate far, and, if so, it can not be hurtful in temporary teeth.

Dr. Grant Molyneaux, Cincinnati, asked whether there was not albumin in the substance of the dentin; we have lime, the inert matter, and the organic matter. The combination of silver nitrate with albumin will produce albuminate of silver if it stands only a few hours. If the deposit is of albuminate of silver we do not understand its action.

Dr. J. S. Cassidy, Covington, said that in case silver nitrate comes in contact with albumin there would be formed albuminate of silver, the combination of which with the moisture in the tooth would produce oxid of silver, which darkens the teeth.

Dr. Molyneaux said that was just what he wanted to bring out; and now the question was whether the albuminate of silver would give us the result that we expect from silver nitrate.

Dr. Dunn said that a gentleman this morning suggested the use of sodium chlorid after silver nitrate; this would produce silver chlorid, which would not darken the tooth.

Dr. W. A. Price said: Not one-thousandth part of the silver put in the tooth under electrolysis remains silver nitrate. Oxygen goes toward the positive pole and meets silver as it goes in. As silver nitrate under cataphoresis it will not form a deposit against the wall of the tooth, unless just at that point it should meet the other atom which would be oxygen.

Dr. Ambler said: In the use of silver nitrate we try to make a deposit the whole length of the root-canal. If you use silver nitrate and know it is a good thing, then why do you use sodium chlorid to destroy that which you have already done?

Dr. Snyder said: We have had the subject of root treatment pretty well discussed, and he regretted that he had forgotten in his paper to mention anything in regard to silver nitrate, but had rather generalized the treatment of the pulp, wishing to make a thrust at careless operators and manipulators. We all try for painless dentistry, but in working out our pet theories we fail to accomplish the desired end. When, for instance, the dentist puts a gold crown on the tooth and leaves a live pulp under a thin layer of dentin, we all know what the result is likely to be. The crown must be removed and the work done that should have been done at first. He was not anxious to be considered a nerve-killer, but we should all look to the probability of the future. He used arsenious acid. Every operation depends upon two things, proper instruments and proper use. Arsenious acid in his hands works well, but in other hands it might not work so well. Carbolic acid in treatment of pulp-canals is good. Whatever the action of silver nitrate should be, it had been a failure in his hands. He finds that the walls of the root-canals near the apex are nearly as sensitive after the use of arsenic as if the pulp were alive. Theories are numerous—not all are bad. We do not want to treat teeth allopathically or homeopathically, but rather eclectically.

The subject was passed, and Dr. W. S. Locke read the following paper:

PHYSICAL CHANGES THAT TAKE PLACE IN TISSUE UNDER PRESSURE.

In this paper it is not my intention to present to you a method of regulating, but if possible some ideas as to the application of force, the effect produced on the tissues of the mouth during regulating, and why we so often fail in our efforts to correct an irregularity.

We hear or read papers written by members of our profession introducing some method or appliances, showing illustrations and models of cases successfully carried through, and the paper will be so beautifully written that the practitioner who has not had experience in this line is led to believe that it is very simple and needs no study whatever. These papers are usually written by men who are well advanced in this branch, and they give you their advanced ideas, little thinking that but few dentists have any understanding as to the first principles of orthodontia.

It is no wonder then that we have so many failures when cases are undertaken by operators who go blundering along, not knowing why they do this or that—not knowing the physiological changes that are taking place in the surrounding tissue, the amount of resistance and force necessary to move a certain tooth, the distance it can be moved with safety within a given time, and last, but not least, they have no idea as to the position the teeth will occupy, or the facial expression of their patient when the case shall have been completed.

Before we place a gold filling in a tooth we have in our mind's eye the operation about as it is to be made. We know the amount of anchorage necessary to retain it in position, and we also know very nearly what the appearance of said filling will be. Now, why is it not possible to see into the future of a regulating case, as we do into the future of a gold filling operation? It is possible, but not without a knowledge of the subject at least the equal of that we have of other branches of the profession. With the proper knowledge it is now possible to correct almost any deformity.

It is supposed that in ancient times irregular teeth were less frequently found than at the present day. The supposed cause is that the ancient races were more uniform in size than those of to-day, and that the intermarriages of the different races of people, having widely different characteristics, were less frequent than now. It sometimes happens that one parent possesses a large frame with big jaws and teeth, and the other small frame with correspondingly small jaws and teeth.

The offspring may inherit the small jaws of one parent and the large teeth of the other, the result being dental irregularity. Sometimes the large jaws of one parent and the small teeth of the other will cause abnormal interstitial spaces between the teeth of the offspring. Now, if it is a fact that these abnormalities are becoming more frequent as time rolls on, it is necessary that our profession be abreast with the times and be able to prevent or correct such cases.

It is first necessary that we shall be familiar with the eruption and calcification of the deciduous and permanent teeth, and in the young child many cases of irregularity can be modified or entirely prevented by the necessary treatment before all the teeth are erupted.

When mechanical force is necessary, the most important feature, in order to be successful, is to know the physical changes that take place in tissues under pressure and the cause of such changes.

When in the act of regulating, pressure is put upon the tooth to move it; the first effect produced is compression of the pericemental membrane on the advancing side of the tooth and a stretching of it on the other. In this compression of the membrane the blood-supply is shut off, and the nerves become irritated. The irritation causes the development of cells known as osteoblasts, the function of which is to break down and absorb that irritated tissue. On the opposite side of the tooth quite the reverse is taking place. Instead of compression there is tension on the membrane. The osteoblasts have been developed for the formation of new alveolar process to close the space left by the tooth.

When a case is presented for correction, and after good impressions are taken, it is well to measure and learn the amount of space occupied by the irregular teeth, the amount of space left in the arch, if any, and the room they will need in their new positions. This can be done, and very accurately. We will take one of the most common of all cases. The eruption of a cuspid outside of the upper arch, with a little space underlying it between the lateral incisor and first bicuspid. The lateral incisor inside of the bite, with space between central incisor and cuspid, but not sufficient to allow it in the proper position. Measure the width of cuspid and lateral; add to that width about one-fiftieth of an inch (I might call it elbow room) for these teeth and each of the adjoining teeth, for it is impossible to leave them in the same crowded condition they were before the case was undertaken. The difference between that width and the space underlying the cuspid, and between cuspid and central, will be the amount of space necessary to provide for.

When force is applied to move a tooth, it should at first be light, as the exact amount of resistance it may give is unknown, and increased a little at each sitting, until the tooth is moving at the desired rate. Should the force be too sudden and strong, there is great danger of setting up an irritation which would cause the breaking down of all the tissue surrounding the tooth, even on the side from which advancement has taken place, as the rate of absorption of process is far greater than that of its formation; besides, the absorption is more rapid at the neck of the tooth, where the pressure is the greatest, and tilting will be the result, which is also likely to cause strangulation and death of the pulp. Again, when force is applied too suddenly, it may move or make very sore the teeth used for anchorage.

In applying force it seems best, when possible, to use the method that will give both positive and intermittent force, for when positive force is used and skillfully applied there is no doubt about the distance or the way the tooth or teeth will move; and when intermittent, is productive of but little pain, no irritation, except at the point of pressure, and it allows the immediate rebuilding of the bone and other tissue in the space just vacated. When constant force is used I find the rebuilding of the tissues far in arrears of the moving tooth, and it will become very loose and extremely sensitive to the touch. No attempts should be made to exert force other than in a direct line. You can not shoot around a hay-stack, even if your gun barrel is bent, nor can you successfully push a tooth in a curved line with but one direction of force. Care should also be taken that the point of resistance from which we exert pressure and the points of resistance and delivery of force are fixed points, which means that rubber bands or appliances of any kind should not be used in such a way as to shift their positions and in that way change the direction of force.

In the early part of August, 1896, the writer was called in consultation with a dentist who had attempted to regulate the teeth of a young lady by the use of rubber bands. The case was an anterior protrusion of both the superior and inferior maxillaries.

The two first bicuspids of the superior maxilla had been extracted and heavy bands drawn around the first molars on each side and over the cuspid teeth, hoping to draw the cuspids back in that way.

The patient was given an appointment and dismissed; she suffered great pain until the day for her return, which was nearly a week, without removing the bands. At this time it was found that the rubber bands had slipped far up under the gums, almost extracting both molars, and drawing them, together with the second bicuspids, forward, closing the space left by the first bicuspids; great irritation had been set up, and all the surrounding tissue was fast absorbing; about half of the palatal roots of both molars were exposed, and the bite separated at least one-quarter of an inch between the incisors.

It was thought best to give the case rest for a few days, that the inflammation might recede, and then push the molars and bicuspids back to their normal positions. This the dentist accomplished in about one month, at which time the patient was put entirely in my hands. Had his appliances been firmly and properly applied this trouble would never have happened. On September 2d I took charge, and completed the case December 22d of the same year, reducing both maxillaries as well as drawing the teeth backward into the proper position. The results of the rubber bands are still to be seen in the mouth and on the models. While on this subject of rubber bands, a little method I use, when I find it necessary to apply them, for retaining them to the crowns of teeth, keeping them from slipping either up or down, is to tie a ligature around the neck of the tooth, place the rubber bands in position, carry the ends of the ligature through the fissure of the crown and tie them tightly to the ligature on the other side of the tooth.

In the positive method there are also many appliances in use that do more harm than good.

In one of our books of reference there is a drawing shown where a first molar and second bicuspid are banded with tube soldered to the buccal side of bands extending from the distal side of the molar past the mesial side of the bicuspid. Through this tube is run a threaded shaft to a cuspid tooth which is leaning forward. The intention is to draw the cuspid to the place of the first bicuspid, and at the same time to force it into the socket. From my point of view this appliance will not accomplish what is claimed for it, and, in fact, will in most cases do that which is least desired. Instead of forcing the cuspid into the socket the cuspid will pry on the bicuspid and molar, tilt them and draw their mesial surfaces from their sockets.

Now, if the bands be soldered together and an eyelet be placed near the necks of the anchorage teeth, with the draw-bar attached so that it will be movable, and to an eyelet at the end of the cusp of the cuspid tooth, the force will exert the same on the cuspid, but only in one direction on the bicuspid and molar without prying them outward. There is in the same work another drawing where two laterals are being forced apart in the same manner. I tried this method several times, each with bad results, and the last time opened the space to such an extent that the teeth had to be ground to bring the occlusion to the proper place.

A moving tooth will not always go in the exact direction intended, and it is best not to fasten too solidly to the anchorage teeth, for the tooth being moved usually holds the lever and is liable to do some damage.

If a cuspid is standing at the right angle and you wish to move it bodily, solder the tube to the cuspid anchorage and allow the anchorage teeth to hold the free end of the bar or the lever, as it were; the tooth will then be drawn bodily into position, and can not possibly rotate, tilt, or injure the teeth from which power is exerted.

It does not seem advisable to require a molar and bicuspid to withstand the resistance of a cuspid, for, while it will often accomplish what is desired, it is likely to draw them from their proper positions; and while they will usually return, if allowed to do so, at once, they will not be fit for anchorage for many months; while if a second molar is attached, or an appliance made that will utilize other teeth to assist, there will be no trouble. Place appliances on the labial side of teeth when possible, unless they are directed across the mouth, as the inner wall of the alveolus is much more dense and will resist a greater strain than the outer wall.

In drawing a cuspid backward it seems best not to attempt to force it too hard against the inner wall until nearly back to the desired position, for the resistance is greater when against this wall, and it forces the anchorage teeth outward toward the outer and weaker one. When the appliance is placed on the outside the force is exerted in the opposite direction, throwing the anchorage teeth against the inner and more solid wall, and the cuspid the outer one, or directly backward ; besides, the appliance is out of the way of the tongue.

In placing regulating appliances in the mouth, I have found it an excellent idea when bands are placed on the teeth to be moved to set them in chloro-percha instead of cement ; the bands can then be warmed and slipped off at any time to make changes or to adjust the retaining appliances without any pain to the patient ; and when bands are used around them for retention they are fitted so that they will slip in position before regulating, taking the impressions and soldering after regulating.

This is alluded to because all who have regulated teeth know how very painful it is to remove cemented bands or to fit retaining appliances after the teeth have been removed.

Since the publication of the paper written by Dr. Case for the World's Columbian Dental Congress, on orthopedia, the attention of the profession has been detracted from the mere moving of teeth to, it might be said, deformities of the face. I have given this subject some study, and it is astonishing what results can be obtained. Not only have I seen results obtained by others, but I had the satisfaction of correcting some cases of deformities, changing and improving the features of the face to the perfect satisfaction of all concerned. In one case especially, the superior and inferior maxillaries were reduced, making quite a pleasant-looking face, out of one which had appeared both homely and repulsive. In the discussion following the paper read by Dr. Case the question came up as to whether or not the bone was actually moved or the roots of the teeth moved breaking down the tissue in front and building it up in the space left by the roots. At that time opinions were greatly divided. The few cases handled by the writer have been watched very carefully to note the changes which took place during the operations, and from what was observed I have come to these conclusions:

When teeth are being moved forward, and all are moving at the same time by the exact same pressure, the outer wall of the process is carried bodily forward, there being no irritation whatever, except at the lingual cervical border, showing that a division of the process is taking place at that point, the inner wall remaining in its original position, the space being filled with new tissue, leaving this wall much thicker than before. If the teeth are carried forward one at a time the process is then absorbed and replaced by new tissue. In retracting the teeth where all are carried together the outer wall is carried with them, while the inner wall is absorbed because of the resistance behind it. Should they be carried backward one at a time the outer wall of this process will not be carried with the teeth, but the new growth of bone will follow each tooth, leaving the process somewhat thicker.

When force is applied to the cutting-edge, say of a central incisor, and drawn inward the fulcrum is at the neck of the tooth, and while the crown moves in, the root moves outward to some extent; but where the crown is pushed outward the apex of the root usually remains stationary, and the tooth moves in. A large proportion of those deformities which appear to be due to the protrusion or recession of the lower jaw will be found after careful study of the face, that the chin is in its proper relative position to the molar prominences and the upper part of the nose, and that the deformity is due to an imperfect position of the submaxillary bone. We can not be too careful in the examining of these cases, and observe just where the deformities lie.

Some months ago a lady presented herself to have some operating done, and a great depression of the upper lip was observed. The deformity was spoken of, and she informed me that she had her teeth regulated about a year before that time, and that before regulating the cutting-edges of the superior teeth closed just inside the lowers. On examining the mouth, it was found that both inferior first bicuspids had been extracted and the inferior centrals were drawn back, or rather tilted backward, under the bite of the superior teeth, leaving a deformity as bad no doubt as before regulating. The lady stated that no impressions had been taken before or after the operation, and that the teeth were extracted and the regulating appliances (which consisted of rubber bands about the teeth) were placed in position within a few minutes after she presented herself for treatment.

Now this goes to show that the mouth was not carefully examined, much less studied, and that no effort whatever was made to better the facial expression, but the object was only to make the teeth occlude in the normal way.

Too much credit can not be given such men as Drs. Angle, Case, Jackson, and others, who have worked hard to build up this specialty and obtain the confidence of patients, while many others are unintentionally and unconsciously working to break it down.

DISCUSSION.

Dr. H. A. Smith, Cincinnati, in opening the discussion, said that in the first place he would take exception to the title of the paper. The changes which were treated of did not come under the head of physics. There is a tendency at present toward correct nomenclature. It is very desirable to call things by their right names, so we can know what is meant. Physics pertains to material things. It is the science of principles obtaining in inorganic nature. In dentistry we are not dealing with inorganic nature, and so the term is misused. Before the rise of modern science physics was made to include vital science, but more recently it is confined to inorganic matter. The writer should have called the paper, "The Physiological Action of Living Tissues Under Pressure." The line dividing the physiological and pathological is very narrow. To produce any change in a living tissue we must produce irritation, and then we have hyperemia, which is pathological. The author says in a certain case the blood-supply is excluded. If this is so, the change would not be physiological; it would be death of the part. Then, to follow the point, the cells which he calls osteoblasts would show effect of pathologic condition.

Another friendly criticism would be toward his misapprehension of the word absorb. He should say resorb. Absorb means to draw in, or suck up as by a sponge, or to take something in which is foreign. Resorb, on the other hand, means to take back; so, in vital action, to resorb is to take back again into the system. These differences in the meaning of words are important, as the careful use of words is always important when discussing scientific subjects. The tissue originally given out by the periodontal membrane is taken back; that is, resorbed. Dr. Goddard, of San Francisco, in "The American System of Dentistry," says, "I think resorb expresses the idea more correctly. I presume we can say correctly that the deciduous teeth are absorbed." Herbert Spencer says, "The temporary organs serve their purpose for a while and then are resorbed."

Many men are doing excellent work on orthodontia, and while things go all right it does not make much difference whether they know much about the action of the tissues under treatment or not; but when anything goes wrong then the necessity for a deeper knowledge becomes at once apparent.

There is much said in the paper in regard to continuous pressure and intermittent pressure. He had been, so to speak, brought up on continuous pressure by means of rubber bands, but in careful hands good results are obtained by both methods. The law laid down by Dr. Farrar was, "If you move a tooth a fraction of an inch at nine o'clock and a fraction of an inch at six o'clock you will not have irritation." Well, if you don't have irritation you won't have resorption.

The amount of resistance at the anchorage must be greater than the force necessary to move the teeth that are to be moved, but need not be much greater, and teeth should not be banded unnecessarily. Dr. Black says, "My conclusion is that it is the resistance of the peridental membrane that settles the amount of force necessary to move a tooth. When we make no progress in moving a tooth, the reason is that the peridental membrane has so much power of resistance that there is no irritation and no absorption of tissue." If some persons can bite with a power of one hundred and eighty pounds and others with only eighty pounds, this fact may explain why in some cases there is less effect produced by the same pressure in the effort at regulation than in other cases.

The subject was passed.

*( To be Continued. )*

